# Strategic partnering to improve community health worker programming and performance: features of a community-health system integrated approach

**DOI:** 10.1186/s12960-015-0041-3

**Published:** 2015-09-01

**Authors:** Joseph F. Naimoli, Henry B. Perry, John W. Townsend, Diana E. Frymus, James A. McCaffery

**Affiliations:** United States Agency for International Development, 1300 Pennsylvania Avenue, NW, Washington, DC 20523 USA; Johns Hopkins Bloomberg School of Public Health, 615 North Wolfe Street, Baltimore, MD 21205 USA; Population Council, 4301 Connecticut Avenue, NW, Washington, DC 20008 USA; Training Resources Group, 4401 Wilson Boulevard, Arlington, VA 22203 USA

**Keywords:** Community health workers, Performance, Communities, Health systems

## Abstract

**Background:**

There is robust evidence that community health workers (CHWs) in low- and middle-income (LMIC) countries can improve their clients’ health and well-being. The evidence on proven strategies to enhance and sustain CHW performance at scale, however, is limited. Nevertheless, CHW stakeholders need guidance and new ideas, which can emerge from the recognition that CHWs function at the intersection of two dynamic, overlapping systems – the formal health system and the community. Although each typically supports CHWs, their support is not necessarily strategic, collaborative or coordinated.

**Methods:**

We explore a strategic community health system partnership as one approach to improving CHW programming and performance in countries with or intending to mount large-scale CHW programmes. To identify the components of the approach, we drew on a year-long evidence synthesis exercise on CHW performance, synthesis records, author consultations, documentation on large-scale CHW programmes published after the synthesis and other relevant literature. We also established inclusion and exclusion criteria for the components we considered. We examined as well the challenges and opportunities associated with implementing each component.

**Results:**

We identified a minimum package of four strategies that provide opportunities for increased cooperation between communities and health systems and address traditional weaknesses in large-scale CHW programmes, and for which implementation is feasible at sub-national levels over large geographic areas and among vulnerable populations in the greatest need of care. We postulate that the CHW performance benefits resulting from the simultaneous implementation of all four strategies could outweigh those that either the health system or community could produce independently. The strategies are (1) joint ownership and design of CHW programmes, (2) collaborative supervision and constructive feedback, (3) a balanced package of incentives, and (4) a practical monitoring system incorporating data from communities and the health system.

**Conclusions:**

We believe that strategic partnership between communities and health systems on a minimum package of simultaneously implemented strategies offers the potential for accelerating progress in improving CHW performance at scale. Comparative, retrospective and prospective research can confirm the potential of these strategies. More experience and evidence on strategic partnership can contribute to our understanding of how to achieve sustainable progress in health with equity.

## Background

Many countries have made significant progress toward achieving the 2015 Millennium Development Goals, particularly in health. According to the United Nations, rates of child mortality and chronic under-nutrition among young children have fallen dramatically worldwide since 1990, and as of 2012, it was estimated that antiretroviral therapy for human immunodeficiency virus (HIV)-infected people has saved the lives of approximately 6.6 million people [1]. The global community is now considering the development priorities and challenges for the next 15 years with a focus on impact, equity and sustainability. An intergovernmental working group has proposed a 2030 sustainable development goal (SDG) for health that aims “to ensure healthy lives and promote well-being for all at all ages” [2]. Large-scale, national community health worker (CHW) programmes in low- and middle-income (LMIC) countries have the potential to extend the reach of inadequately resourced health systems to vulnerable and under-served populations and thereby improve service access with equity by 2030.

In light of this potential, CHWs and CHW programmes are experiencing a resurgence of interest and are likely to continue to attract national and international attention and investment in the next decade and beyond [3]. The renewed interest in these programmes can be traced, in part, to an increased focus on the achievement of universal health coverage in LMICs, which is constrained by persistent health workforce challenges in countries with weak health systems, particularly those in sub-Saharan Africa [4–8]. There is robust evidence that CHWs, a heterogeneous cadre of frontline health workers operating in a diverse set of countries and contexts, can improve people’s health and well-being [9].

CHW programmes, however, face many challenges. Political endorsement is often weak, financing is problematic, oversight and technical support are fragmented, a common and well-funded research agenda has been lacking and the evidence base on proven strategies to enhance and sustain CHW performance is modest [4, 10–14]. Nevertheless, policy makers, programme managers, public health practitioners and other stakeholders need guidance [5, 15] and practical ideas for how to support and retain CHWs in large-scale programmes. New ideas may emerge from the recognition that CHWs function at the intersection of two dynamic and overlapping systems – the formal health system and the community. Each system typically supports CHWs; their support, however, is not necessarily strategic, collaborative or coordinated.

In this paper, we explore an integrated approach on how to improve CHW programming and performance in countries with or intending to mount large-scale CHW programmes: a strategic partnership between communities and health systems. Drawing on multiple information sources, we identified four strategies that provide opportunities for increased cooperation. For each strategy, we discuss different aspects of implementation and accompanying challenges and opportunities. We also propose a learning agenda to inform and promote further reflection and discussion among policy makers, managers of health and community-based programmes, practitioners and other CHW stakeholders. We conclude that simultaneous implementation of these four strategies, combined with research on the effectiveness of this integrated approach, could contribute to large-scale, national CHW programmes reaching their full potential, thereby promoting accelerated progress toward achieving global health and development goals and universal health coverage with equity [7, 16–19]. Key terms that we use in this paper are defined in Table 1.

**Table 1:** Key definitions

Keywords	Definition
*Community health worker*	A health worker who receives limited standardized training outside the formal nursing or medical curricula to deliver a range of basic health, promotional, educational and mobilization services and has a defined role within the community system and larger health system. This label applies to a heterogeneous cadre of frontline health workers operating in a diverse set of countries and contexts. For example, in some countries, a CHW may be a full-time, paid employee of the government, while in other countries, he or she may be an unpaid, full- or part-time volunteer supported by an NGO. Both types of CHW may be present in the same country.
*Health system*	All people, institutions, resources and activities whose purpose is to promote, restore and/or maintain health [93]. The health system includes NGOs working in concert with the public and private sectors to provide services and achieve these goals.
*Community*	A social group comprising both kin and non-kin social networks that share a sense of connectedness – through shared values, common interests and/or adherence to norms of reciprocity – and which perceives itself as distinct in some respect from the larger society in which it resides [95]. Potential sources of support for CHWs in the community include both public sector and civil society entities, such as village or neighbourhood health committees, religious leaders and faith-based organizations, social support associations, community-based organizations, multi-sectoral organizations, political and governance groups and women leaders.
*CHW support*	Quality CHW programming comprises a wide range of support activities that explicitly target CHWs and are undertaken by a range of actors in the health system and the community. We have subsumed these support activities, for both the health system and the community, under three common rubrics: technical support, social support and incentives. Technical support includes efforts to design well-functioning CHW programmes that deliver, through sound implementation and management, high impact, evidence-based interventions, monitor adequacy of implementation and evaluate effectiveness. Social support includes fostering partnerships, strengthening linkages with various health and community system actors and entities that can support CHWs in their efforts and providing opportunities for CHWs to interact and support one another. Incentives encompass non-financial, in-kind and financial inducements (including salaries) that are commonly used to motivate CHWs to enhance and sustain their performance.
*CHW performance*	We define CHW performance in relation to the specific roles and responsibilities of CHWs in a given context in three ways: outputs, outcomes and impact. *Outputs* are proximate measures of performance that occur at the level of the individual CHW. Some are indirect measures, such as cognitive/psycho-motor (for example, knowledge and skills acquisition) or affective (for example, self-efficacy/self-esteem, confidence or personal satisfaction) CHW-level changes, while others are direct behavioural measures that occur at the interface of CHWs and clients, such as absenteeism, the quantity and quality of service delivery, responsiveness to clients and productivity. Attrition and advancement are measures of CHW developmental changes over time. *Outcomes* are intermediate measures, defined as CHW-attributable changes that occur among individual clients (for example, healthcare-seeking behaviour or health-promoting behaviour in the home), as well as effects on communities and health systems (for example, changes in social cohesion or cost savings to the health system, respectively). *Impact* refers to more distal measures, defined as CHW-attributable changes in health (for example, morbidity and mortality) at the population level.

In theory, fostering a robust community-health system partnership, in which both entities work strategically and collaboratively toward a common end, offers the potential to strengthen CHW programming and enhance CHW performance. Our rationale is as follows. *First*, increased strategic cooperation could produce performance benefits for CHWs that outweigh those that either entity could produce separately. By combining their support efforts in a purposeful way, health systems and communities could provide better value to CHWs by revisiting, in novel ways, traditionally independent approaches to improving performance and by identifying and testing innovative solutions in settings where resources are and will continue to be limited.

*Second*, a more strategic alliance permits communities and health systems to take better advantage of one another’s assets and capabilities and to coordinate their efforts around a shared objective of mutual benefit: higher performing CHWs. Through this collaborative, dynamic relationship, both entities can share more information and appropriate technologies, accommodate their needs, take action more quickly and build trust [20]. The synergies created by this increased cooperation can help maximize the efficient use of available resources.

*Third*, heightened trust, combined with demonstrable results in improving CHW performance, may lead to positive spillovers to other areas of public health that require optimal collaboration between the formal health system and communities (for example, water and sanitation and malaria eradication). Successful partnerships, however, are not born – they must be made. They require a serious commitment to good governance (explicit mutual responsibilities and accountabilities), a demonstrated willingness by both parties to work in tandem toward a common objective and flexibility. Each party must contribute the requisite time, attention, skills and resources necessary to ensure success. Not all partnerships are identical – the best ways of working together must be tailored to the diverse needs and characteristics of communities and health systems.

*Fourth*, there are examples in the literature of successfully functioning CHW programmes that report the presence of combined support from the community and the formal health system [21, 22]. The extent to which this combined support was intentionally pre-designed, however, is not well-documented. Moreover, the global health community is increasingly recognizing the need for constructive collaboration between CHWs and professional health workers in the public and private sectors to strengthen health systems, improve the delivery of essential services [23, 24] and ensure an effective continuum of care. The collaborative partnership in support of CHWs that we propose is consistent with observations on the rapid spread of child survival, maternal health and HIV/acquired immunodeficiency syndrome (AIDS) interventions in participatory democracies [7, 25, 26].

## Methods

Our identification of a minimum package of simultaneously implemented essential strategies arose primarily from a sequential process that drew upon three key events: (1) a year-long (2012–2013) exercise to synthesize the evidence on strategies to enhance CHW performance in large-scale, national programmes [14]; (2) a review of evidence synthesis records; and (3) a series of author consultations. We consulted additional CHW-relevant documentation since the completion of these events. In this section, we briefly describe each event, and we specify our inclusion and exclusion criteria for the selection of strategies.

*First*, all the authors were participant–observers in the evidence synthesis exercise. The synthesis included a structured review of the available research (both published and gray) on the support (technical, social and motivational) that health systems and communities provide to CHWs. Although the primary focus of the synthesis was large-scale, national programmes, the review included documented experiences from small- and medium-size efforts in the public, non-governmental and private sectors.

The principal finding of the review was that the relationship between CHW support and performance (change at the level of individual CHWs, the clients they serve and the broader population) is not well-understood. This is not because rigorous studies of the support–performance relationship have demonstrated a lack of effect; rather, researchers have not commonly raised or properly investigated questions about which interventions are most likely to improve and sustain CHW performance at scale. It is important to note that research challenges and funding restrictions have limited learning about this relationship. Consequently, the synthesis exercise was unable to produce sufficient evidence to successfully validate the review’s central hypothesis – *that the combined effect of community and formal health system support activities on improving CHW performance is greater than the effect of either alone*. Each of the authors served as a member of one of three evidence review teams (ERTs).^1^

The organizers of the review disseminated the findings during a U.S. government-sponsored evidence “summit” – a facilitated discussion with approximately 150 international experts (2012). Experts included leaders of large-scale, national CHW programmes, managers of non-governmental organizations (NGOs), specialists who monitor and evaluate community-based programmes utilizing CHWs and decision makers who formulate national policies related to CHW programmes in Asia and Africa. All the authors participated in the 2-day summit, which provided them with an opportunity to consider the available evidence in light of informed opinion about options for enhancing CHW performance. Detailed information on the evidence synthesis exercise, including the literature search strategy, the work of the ERTs, the evidence summit event and other aspects of the synthesis exercise is available at this link: http://www.usaid.gov/what-we-do/global-health/chw-summit.

*Second*, at the conclusion of the literature review–summit exercise, all the authors independently read the three commissioned ERT evidence synthesis papers. Each paper described the methods and findings of the structured reviews, by source of support (that is, the community, the health system and the health system and community) [21, 27, 28]. In addition, the authors consulted the written proceedings from the summit, which captured important commentary and the recommendations of international experts.

*Third*, although the literature review–summit exercise did not yield a definitive conclusion about the best way to improve CHW performance, the authors agreed that the year-long exercise provided many practical examples and new ideas about how communities and health systems might work together better to improve CHW performance in large-scale programmes. Consequently, the authors met on multiple occasions over several months to identify, through group discussion, a limited number of strategies that they believed offered promising opportunities for productive strategic partnership. Candidate strategies had to meet the following inclusion criteria: (1) enhanced cooperation between health systems and communities was reasonable and promising based on the literature and expert opinion, (2) implementation was feasible at sub-national levels over large geographic areas and among vulnerable populations in the greatest need of care and (3) collective action would address traditional weaknesses in large-scale CHW programmes.

*Fourth*, in addition to their review of the three commissioned synthesis papers, and the commentary and recommendations of the summit experts, the authors consulted several key documents on large-scale, national CHW programmes published since 2013, as well as other literature relevant to the strategic components of the integrated approach. Preliminary ideas about the choice of strategies to be included in the approach were influenced by this additional research, further discussion among authors and the solicited views of experts. For example, we excluded from consideration certain strategies that may be critical to the success of large-scale CHW programmes but which require strategic partnership between different sets of actors.

For example, large-scale, national CHW programmes often fail to achieve their desired outcomes because they are inadequately resourced, both in the short and long term [29]. With some notable exceptions, government, community and donor financing of large-scale CHW programmes have all encountered important limitations [29]. In response to these limitations and stimulated, in part, by recent interest in universal health coverage [30] and the prospects of increased domestic resource mobilization for health [31], advocates are increasingly calling for more and better strategic partnerships between governments and donors to ensure adequate financing of large-scale CHW programmes [3]. We decided that these kinds of complementary partnerships for other critical CHW-relevant strategies fell outside the scope of this paper.

Different authors took responsibility for crafting each of the four strategies, drawing upon different combinations of source materials. All authors reviewed and commented on all the strategies. All authors reviewed and commented on multiple iterations of the manuscript.

## Results and discussion

Our minimum essential package includes four strategies that met our inclusion criteria. We propose simultaneous implementation of all four strategies as an integrated approach. We postulate that the CHW performance benefits resulting from the simultaneous implementation of these strategies could outweigh those that either the health system or community could produce independently. The four strategies are as follows: (1) joint ownership and design of CHW programmes; (2) collaborative supervision and feedback; (3) a balanced package of incentives, both financial and non-financial; and (4) a practical monitoring system that incorporates data from communities and the health system. For each strategy, we present implementation considerations and discuss a range of accompanying challenges and opportunities. We also propose a formal research learning agenda, the implementation of which can generate new knowledge about strategic partnering to guide continuous learning and problem-solving. We also discuss some of the limitations of our process in identifying the component strategies of our integrated approach.

### Strategies

#### Joint ownership and design of CHW programmes

##### Implementation

Joint ownership of CHW programmes begins at the national level but is expressed operationally at the community level over large geographic areas to achieve scale. National-level dialogue can produce a broad framework for how collaboration might work at the community level; however, with increasing health sector decentralization, local leaders and stakeholders are likely to have greater freedom to adapt and adjust normative guidance from the centre to meet the needs of local conditions. This adaptation process can increase accountability of the health system, improve access to care and increase consumer satisfaction [32, 33]. Decentralization provides an opportunity for communities and health systems to find innovative ways to design sound programmes for the benefit of CHWs and the communities they serve.

Collaborative CHW programme design requires close coordination among many partners: ministries of health, district-level officials, donors, NGOs and other implementing partners, relevant private for-profit and not-for-profit representatives, professional associations and regulatory bodies and representatives of the community (for example, advocacy groups, civil society representatives and local community leaders). It has been reported that programme “co-creation” holds the promise of enhancing shared ownership from the outset [34]: mutual responsibilities can best be linked efficiently in assessing the needs of the community; determining CHW roles and functions that respond to these needs; identifying selection criteria; developing strategies for recruitment, training and retention; planning for how to enhance CHWs’ relationships with other providers, in both the community and health system, and linking them to different service delivery systems; determining career opportunities for CHWs within both public and private sectors; and developing a system for routine measurement and accountability for performance improvement informed by both health system and community perspectives [15].

Decision makers and practitioners looking for pragmatic approaches for how communities can collaborate with health systems might consider the following examples, drawn from various countries:

- Early discussions with community leaders to engage in the CHW selection process [35–38] and to agree on ways to provide support to CHWs [39] to facilitate their work and enhance motivation.
- Asking a sample of community members simple standardized questions about their salient health problems, their needs and potential solutions for addressing their problems and satisfying their needs [40].
- Community and village health committees, oversight and management bodies and CHW advisory groups can complement health system support of CHWs in designing appropriate programmes [41–44].
- Community meetings held at the time of the initiation of the CHW programme to clarify the role of the CHW [45] may foster “community embeddedness” [7, 34, 46].

##### Challenges and opportunities

A move toward shared ownership and design of CHW programmes may represent, in certain settings, a substantial change in existing power dynamics within and among families, communities and local governance structures. Resistance from established interests in response to proposed changes at the community level has been well-documented [47, 48]. Where mutual respect and trust among CHWs and key actors in the community and health system have been absent or weak, building this relationship may have to be a first priority [49]. For the community, action may be required to mobilize appropriate leadership and participation that includes community leaders or members who are accountable to the entire community and who can work with representatives of the formal health system [49].

Also, the community may need to establish structures and identify and implement procedures for collaborating with CHWs, if they do not already exist. From the most peripheral health facilities in rural areas to the most advanced facilities in urban areas, cooperative mechanisms that support health system–community collaboration are likely to be needed to facilitate community assessments and discussions with community leaders that promote inclusive decision-making by individuals and groups. In many countries, women figure prominently as CHWs [50]; how their need for recognition, mobility and reimbursement is addressed will influence women’s overall sense of autonomy and agency, affect the power dynamics within families and may have implications for economic growth as more women enter the workforce.

#### Collaborative supervision and constructive feedback

##### Implementation

Supervision of CHWs has been a persistent weakness of large-scale, national programmes, and accountability to communities has often been lacking. Analysts often report that supervision of CHWs, which has traditionally been provided by the health system alone, is too infrequently implemented to be useful or effective [38, 45, 46, 51–57]. Evidence from the literature on supervision of healthcare providers at peripheral service delivery locations, including CHWs, suggests that supervisors seldom travel to supervise community-level staff because of other duties in patient care and administration, a lack of funds to cover transport costs for field visits or a general disinclination to carry out this responsibility. The literature suggests that more frequent supervision does not necessarily result in better performance or improved patient care, often because supervisors lack skills in problem-solving and coaching. Finally, when supervision does occur, rather than being supportive, it is often limited to fault-finding.

Although there are few examples of where collaborative supervision has been achieved over large geographical areas and institutionalized on a large scale, it has been reported that timely and effective collaborative supervision can boost CHW status in the community [58]. A recent review of the peer-reviewed literature on supervision of peripheral health workers, including CHWs, identified several promising approaches for strengthening supervision [59], all of which could benefit from collaborative engagement by actors from both the community and the health system.

- Meeting of the supervisor with groups of CHWs to solve problems and set goals;
- Engaging stronger peers in support of weaker ones through peer assessment, on-the-job training and mentoring;
- Monitoring and assessing CHW and other health workers’ performance by communities through, for example, consumer reporting on provider performance [60] or verification measures associated with performance-based financing programmes [61];
- Sharing the results of periodic self-assessments with a supervisor, possibly by recording them on a cell phone and transmitting them to the supervisor; and
- Using supervisory checklists coupled with recommended actions to address the individual and system performance issues.

##### Challenges and opportunities

Although engaging communities in the supervisory process is intuitively appealing, we were not able to identify any published examples where such collaborations have been institutionalized in a large-scale CHW programme in a particular country. For collaborative supervision to be successful, and considering the current state of supervisory practice, community leaders and their health system partners may need to start afresh. CHWs need practical, feasible and supportive supervisory approaches that avoid the pitfalls of routine supervision as it is commonly practised by health systems and that can be tailored to the local context and the diverse capacities and needs of communities. A starting point may be to lodge a new approach at the lowest administrative levels of the formal health system in partnership with communities. Such an approach could contribute to higher quality services provided by CHWs, improved health-promoting behaviours in the household and greater health system utilization, resulting in improved health of the population. Smart phones and other relatively inexpensive communication media may provide opportunities for continuing education and connecting CHWs and supervisors from the health system and communities on a more regular basis [62].

#### A balanced package of incentives, both financial and non-financial

##### Implementation

Communities and health systems can marshal their respective resources and coordinate their efforts in providing inducements for improved CHW performance. There is a promising body of evidence suggesting that performance can be improved when CHWs receive both financial and non-financial incentives linked to performance [22, 26, 63–65]. For example, Afghanistan, Indonesia, Nepal, Rwanda and India all have national programmes that use volunteer CHWs who generally receive some combination of both kinds of incentives [50]. Both the health system and the community typically play complementary roles in providing these incentives.

Rwanda’s government-sponsored Community Performance-Based Financing scheme compensates approximately 44 500 CHWs for their performance (evaluated every quarter) by contributing financial incentives to 445 cooperatives of which they are members (approximately 100–250 members per cooperative) [66]. The scheme pays CHWs for providing selected MCH services and rewards them for the quality of their reporting and management practices [61]. The health system also provides CHWs with non-financial incentives, in the form of training to further develop their skills and motivate them to provide high-quality services [66]. Communities contribute to CHW performance through their participation in and contribution to income-generating schemes mounted by the cooperatives.

The Accredited Social Health Activist (ASHA) in India receives from the health system performance-based incentives for a range of interventions on a fee-for-service basis (for example, $10 for facilitating an institutional delivery and $2.50 for facilitating a child’s completion of the vaccination schedule) [50]. In certain states, ASHAs may also benefit from cash rewards, recognition in the media and career development opportunities. Village health and sanitation committees, comprising village residents, provide non-financial support to ASHAs in helping them to carry out their activities.

Financial incentives can take many forms: a fee in exchange for service, as exemplified in the cases of Rwanda and India, special stipends, allowances and salary. Questions related to CHW salary remuneration – including whether, for whom, and how much – are a prominent theme in the literature on CHW policy and programming [38, 63, 67–69] and a much-debated topic. Several large-scale, national programmes have provided a salary for full-time, or near full-time, work (for example, Ethiopia and India) [50]. Some have argued that community volunteers who are expected to work more than a few hours per week should be compensated in some way as there is little evidence that volunteers can or will sustain an adequate level of effort [70]. Many volunteer CHW programmes have high attrition [71], in part because of the high-opportunity costs of CHW time and effort [55, 72–75]. High turnover usually results in increased costs associated with replacement recruitment and training and impedes progress on programme performance because of delays in filling vacancies. Gender equity issues may emerge if women are asked to voluntarily provide these services. As an alternative to a paid-only or volunteer-only scheme, Nepal has successfully mounted a “mixed” model, which includes both full-time paid CHWs and part-time volunteers [3].

In 2010, the World Health Organization (WHO) released a guideline document that contained a series of recommendations on how to increase access to health workers in rural and remote locations [19]. Its report, similar to our review, drew upon both an extensive literature review and informed opinion. Although aimed largely at facility-based healthcare providers, many of the recommendations are relevant to CHWs. Of particular note, the report recognizes the importance of non-financial incentives, including better living conditions, a safe and supportive working environment, outreach support, career development programmes, membership in professional networks and public recognition for improving their attendance at work, performance and retention.

Traditionally, the community has been the primary source of non-financial and in-kind incentives. Health systems, however, also can provide non-financial incentives. In different settings, health systems provide the following kinds of non-financial incentives to frontline health workers, including CHWs: preferential access to healthcare services for the worker and possibly his or her family, provision of healthcare services at reduced cost (or free), career growth opportunities, continuing education, mentoring, performance reviews, ensuring an adequate supply of commodities and equipment needed by CHWs, recognition of outstanding performance and providing visible examples of a CHW’s special status (such as identification cards with photo, uniforms or bicycles) [39, 51, 58, 76–78]. In Honduras, a letter from the Secretary of Health thanking the family of a community volunteer (“monitora”) for its generosity was deemed to have important intrinsic value for the CHWs [79].

##### Challenges and opportunities

There is no “one size fits all” approach to combining financial with non-financial incentives, which are likely to be provided by multiple sources in the health system and the community. Rather, there needs to be a menu of blended options, with explicit advantages and disadvantages that CHW programme leaders and practitioners can consult when deciding upon an appropriate package of incentives. This need is not unique to CHWs: all healthcare professionals could benefit from such a menu of options. There has been little effort to date, however, to extract from the available literature what might constitute a suitable set of options, and to comparatively test alternatives, although two recent publications have sought to provide a comprehensive review of the available evidence related to CHWs [77, 80].

There is no conclusive body of evidence about CHW financial and non-financial incentives, including salary remuneration, to guide programme design and implementation in every setting. This is understandable considering that the evolution of CHW programmes varies from one country to another. Intuitively, CHWs who feel their efforts are appropriately compensated and recognized, and who perceive long-term value in their role as CHWs, may be better placed to provide higher quality, essential services to the populations they serve. Those designing effective CHW programmes should aim to optimize the impact of the complementary roles of the community and the health system in identifying and providing a contextually appropriate incentive package that has benefited from the deliberations of key local stakeholders.

#### A practical monitoring system incorporating data from the health system and community

##### Implementation

Whether the objective is to monitor the performance of individual CHWs or the performance of the CHW programme as a whole, informed judgments require high-quality data that are routinely collected, analysed appropriately and utilized for decision-making [80]. A practical, simple monitoring system that incorporates data from both the community and the health system can improve both accountability and CHW performance. In our opinion, such a system should include two important elements: one that monitors and assesses CHW performance and one that monitors the availability of essential materials, supplies and commodities for CHWs.

A CHW performance monitoring system provides the basis for early detection of needs and problems, continuous learning and identification of responses to individual and health system constraints. Traditionally, this kind of information has been collected more frequently by the health system through health record reviews, supervisor observations and, on occasion, home visits. However, community leaders and community groups can also be active participants in monitoring CHW performance, as experiences from Uganda and India have demonstrated [60, 81]. Their actions can enhance the community’s governance of CHWs. Feedback from CHWs on their own performance, on overall CHW programme performance and on the performance of peers could provide additional valuable sources of information. The monitoring system could also include data about the effectiveness of referral processes, which is a key factor in connecting the community and the health system.

Linking improvements in CHW performance to important health outputs and outcomes, whenever possible, could help to justify continued investment in the CHW programme and identify areas where alternative CHW programme strategies might be required to improve performance. Opportunities for increasing the effectiveness of monitoring systems have increased with the availability of low-cost mobile technology; for example, handheld devices can supply real-time data for decision-making and offer greater accessibility to supervisors. A recent systematic review of the literature on CHWs and mobile technology found some promising evidence that mobile tools can help CHWs to improve both the quality and efficiency of the services they provide [62]. Another review found, however, that there are few studies that have demonstrated an impact of portable electronic devices on clinical outcomes [82]. It also reported a dearth of such applications for services operating at scale [82].

Routine shortages of supplies and commodities erode the capacity of CHWs to deliver appropriate services, contribute to low demand for CHW services and thereby negatively impact performance [57]. These shortages also contribute to CHW frustration and job turnover. Community support for CHWs could be further mobilized if members could be engaged to track the availability of essential supplies. Arrangements with private sector logistics systems to ensure the availability of critical commodities in the community may be possible where public sector systems are unable or unwilling to include CHWs in their distribution networks. Ideally, such a monitoring system should be synchronized with existing health management information systems (HMIS).

##### Challenges and opportunities

Existing mechanisms for assessing the performance of CHWs in large-scale, national CHW programmes are not well-developed. We identified four types of challenges for practical monitoring systems that aim to link communities and health systems in such efforts. *First*, we need more and better data on CHWs – who they are, where they are and what they do. Community and health system actors can work together to collect these data and ensure they are incorporated into the national human resource information system (HRIS), a component of the national HMIS. *Second*, those in the health system may resist implementing such systems, particularly where a functioning HMIS exists. However, most HMIS are usually facility-based; they tend to track number of services provided rather than key processes – such as quality of care or the functioning of supervisory and logistical support systems – are often under-utilized (with the more remote service sites having the weakest systems) and are a passive means of data collection. The monitoring system should be presented as an extension of the current HMIS, a means of enhancing its utility by addressing some of its limitations.

*Third*, in circumstances where more active, continuous monitoring of CHW performance is possible, the quality of the data for adequately assessing CHW performance or identifying trends may be imperfect at the beginning. Health systems and communities may initially lack the capability to collect this information and to use it to make timely decisions to improve services at the local level. These constraints, coupled with lack of funds, will require time to build capacity through learning by doing, identify incentives for routinely collecting and using data to strengthen CHW programmes and leverage health system and community assets to support and sustain such systems. Mapping techniques should be considered for identifying the spatial distribution of facilities, services and personnel. Such techniques hold considerable promise for focusing attention on the total market for healthcare in large geographic areas.

*Fourth*, periodic surveys and external assessments and evaluations can provide crucial information related to CHW performance and are therefore alternatives to a continuous monitoring system. These activities, however, tend to be costly, externally designed and infrequent and organized to provide only periodic feedback to the health system at a higher level. They also require more sophisticated data management capabilities than a routine performance monitoring system. Mobile reporting on performance is promising but is still a long way from offering significant relief from the challenges CHWs and those monitoring their performance commonly face [82]. Some analysts have called for more attention to “structured experiential learning”, by which implementers complete rigorous, real-time tracking of important aspects of programme performance, with tight feedback loops and continuous attention to addressing performance problems [83]. One option is to judiciously stage and combine external periodic assessments with continuous data collection, learning, feedback and adjustment.

Table 2 summarizes the various challenges and potential responses, by strategy, described in this section.

**Table 2:** Summary of illustrative challenges and potential responses, by strategy

Strategy	Illustrative challenge	Potential response
1. Joint ownership and design of CHW programmes	Overcoming resistance from established interests	• Capitalize on health sector decentralization to build mutual respect and trust among CHWs and the many community and health system actors
		• Mobilize community and health system leaders accountable to the entire community
		• Establish explicit structures and processes for community and health system collaboration
2. Collaborative supervision and constructive feedback	Engaging communities in the supervisory process and institutionalizing the approach	• Build a new model of collaborative supervision from the ground up that responds to local context and takes advantage of community and health system assets
		• Enlist health system supervisors as mentors of community counterparts through on-the-job training and learning by doing
		• Encourage and facilitate community reporting on CHW performance that engages health system supervisors in design and implementation
		• Explore the potential of relatively inexpensive mobile communication media, keeping in mind its limitations
3. Balanced package of incentives	Identifying the proper mix and sources of financial and non-financial incentives	• Develop a menu of options with explicit statements of advantages and disadvantages of each
		• Test and modify different approaches to optimizing the impact of the complementary contributions of communities and health systems
		• Maximize the full potential of non-financial incentives originating in both communities and health systems
4. Monitoring system	Resistance by health system to support continuous monitoring in the presence of a functioning HMIS	• Present monitoring system as an extension of HMIS, as a means of enhancing its utility by addressing its current limitations
		• Adopt a long view; build capacity through learning by doing; find incentives for data collection and use; leverage community and health system assets to support and sustain
	Adequate implementation and ensuring data quality	
		• Judiciously stage and combine external periodic assessments with continuous data collection, learning, feedback and adjustment
	Overcoming preference for periodic surveys and external assessments/evaluations	

### Limitations

Our exploration of the potential of a community health system strategic partnership to improve CHW programming and performance faced several limitations. *First*, we drew our strategies, in part, from three commissioned reviews of the literature, which varied in their approach, quality and rigour. In some cases, it was difficult to ascertain with certainty which recommendations in each report were based on research evidence, which on expert opinion and which on experience. Also, it was not always clear whether assertions and recommendations were drawn from large-scale, national programme experience alone.

*Second*, we drew our strategies, in part, from expert opinions expressed by those who attended the 2-day evidence summit. Those who attended and participated served as a convenience sample of experts: they were not necessarily representative of the broader population of expertise on CHWs around the world. *Third*, our choice of strategies was based, in part, on the informed opinion of the authors. Ideally, a wider vetting of our selections would have increased confidence in their face validity.

We recognize that our strategies are not all-inclusive. No doubt others that might be considered critical to CHW programme success and offer the potential for strategic community-health system partnership could be considered. We believe the four we propose, however, do conform to our criteria and are therefore a starting point for further reflection and inquiry. Because of the weaknesses inherent in each of our data sources, we consulted additional documentation, primarily from the peer-reviewed literature, and attempted to triangulate our information from all these sources to develop a plausible approach for a community-health system strategic partnership. We acknowledge the exploratory nature of this exercise: all the co-owned, co-managed strategies we propose require further investigation and verification. Finally, we recognize the wide diversity of contexts in which CHW programmes operate; consequently, our proposed integrated approach to partnership would need to be adapted to local circumstances.

### The way forward: a learning agenda

There is still much to learn about the viability of strategic partnerships between communities and health systems to improve programming and optimize CHW performance. Studies from large-scale, national CHW programmes on the effects of a strategic partnership approach are lacking. There are different yet complementary ways to pursue a learning agenda about enhanced partnering for improved CHW performance.

*First*, case studies from the literature on government–community partnerships for strengthening programmes in sectors other than health in LMICs, and possibly in certain settings in industrialized countries, can provide new insights about how to structure, manage, execute and evaluate partnerships for CHWs. *Second*, how to move from joint ventures in small-scale projects in local areas to partnerships in large-scale programmes at the national scale is not well-understood; consequently, the literature on diffusion of innovations [84] and scaling up may also provide complementary lessons [85].

*Third*, action research can increase our understanding of the features of a successful partnership that are required for success, as well as identify novel approaches and unexpected consequences, both positive and negative [86]. Action research sacrifices the control traditionally associated with classic research; however, it does provide opportunities for responsiveness, experimentation and innovation that are not possible with more classic approaches to research [86]. Decision makers can use the real-time data generated by this kind of research to identify problems and to take corrective actions to improve execution and enhance future planning. Good documentation of implementation provides important data about not only the adequacy of effort but also the conditions necessary for transferability of these experiences to other settings [87, 88].

*Fourth*, in certain situations and under certain conditions, time series research designs, or those that use systematic variation to learn while scaling up, such as stepped-wedge designs [89], might be feasible and effective in capturing the complex dynamics involved in large-scale programme expansion. Another approach is to mount small-scale, quasi-experiments that can produce insight and evidence during implementation.

*Fifth*, our proposal of a minimum package of essential strategies begs the question of cost. Although CHW programmes are inexpensive if narrowly viewed in terms of cost per service or per CHW [90, 91], the net aggregate of programme costs can be substantial for a government’s limited health budget, particularly when the full cost of managerial and logistical support operations and other initial and recurrent costs are included [2, 13, 29, 70]. One recent estimate of the average yearly expenditure for a CHW programme, based on 1 CHW for every 650 rural inhabitants in the sub-Saharan Africa region, was $3584 per CHW [3]. A similar exercise would be required to cost our proposed minimum package.

*Sixth*, broader efforts to strengthen health system and community performance can have indirect effects on strengthening CHW programming and ensuring sustainability at scale [92]. High-performing health systems with sound governance, adequate financing, well-organized service delivery, a capable and well-deployed health workforce, sound information systems and reliable access to a broad range of medical products and commodities can reinforce CHW-specific programming [93]. Similarly, competent communities could contribute to better CHW performance through sound governance of community resources, promotion of inclusiveness and cohesion, engagement in participatory decision-making and mobilization of local resources for community welfare [94]. More research is needed to understand the contribution of these systemic inputs, processes and activities to improved performance relative to more targeted programmatic interventions. Some concrete actions to move this learning agenda forward are described in Table 3.

**Table 3:** Recommendations for advancing the learning agenda on CHW performance

Action	Actor	Recommendation
Comparative analysis	Global health community	The global health community could facilitate decision-making at country level by reviewing and synthesizing analogous partnership efforts from other countries, disciplines, and sectors – both successes and failures. A short list of applicable insights could contribute to more creative, “out-of-the box” thinking and experimentation to guide planning.
Retrospective analysis	Countries	Countries that are taking CHW programmes to scale may wish to conduct a retrospective landscape analysis of both successful and unsuccessful partnership experiences that have occurred in different districts and communities in their country. Even though many of these experiences are likely to have occurred in small-scale, NGO-managed projects, there may be important lessons that could be tested at scale. Partnership experiences in sectors other than health, such as agriculture or education, may prove to be a rich source of innovation.
Prospective analysis	Countries	Countries also should look for opportunities to develop and institutionalize a prospective, continuous learning and problem-solving process that is tailored to the unique history, dynamics and context of the specific programme. Action research can provide real-time data for continuous adjustment and quality improvement, and it can also provide important information about programme processes that can inform subsequent impact evaluations.

## Conclusions

Our intent was to translate knowledge, from both the literature and experts, into some provisional ideas about improved practice. As CHWs function at the intersection of two dynamic systems, we believe that more strategic cooperation and shared learning between communities and health systems offer the potential for progress in improving CHW performance at scale. There is a growing consensus that CHWs are likely to be key contributors to the achievement of a sustainable development goal for health and universal health coverage in LMICs in the years to come [4, 70]. They are no longer viewed as a temporary solution to persistent and significant health workforce challenges or a second-best service delivery approach [5, 6]. We recognize that the potential for greater efficiency to be realized through an integrated approach to supporting CHWs, building trust between health systems and communities and creating positive spillovers for public health through strategic cooperation must be balanced against the labour-intensive practice of developing and sustaining good partnership practices. The four co-owned, co-managed strategies that we present in the form of an integrated approach demonstrate how the movement toward a more deliberate and efficient partnership between communities and health systems will require careful reflection, collaborative leadership, joint decision-making and action and mutual accommodation.

Lessons learned from analogous partnership activities in other countries, disciplines and sectors; within-country retrospective stock-taking exercises; and prospective action research on the most relevant and feasible cooperative strategies for enhancing CHW performance provide opportunities for continuous learning and problem-solving for better partnering in support of CHWs. Advancing this learning agenda – and encouraging countries and donors to support such activities – will be an important step in helping large-scale, national CHW programmes reach their full potential. Robust CHW programmes can contribute to accelerating progress toward achieving sustainable improvements in coverage and health with equity. Our hope is that this paper will encourage further discussion and generate new ideas about efficient and effective ways to improve CHW performance.

## Abbreviations

SDG: Sustainable development goal
CHW: Community health worker
LMIC: Low- and middle-income country
HIV: Human immunodeficiency virus
AIDS: Acquired immunodeficiency syndrome
ERT: Evidence review team
NGO: Non-governmental organization
WHO: World Health Organization
HMIS: Health management information system

## Footnotes

1 ERT 1 examined the contributions of communities to CHW performance, ERT 2 examined the contributions of health systems to CHW performance and ERT 3 examined the combined contributions of communities and health systems, where they existed.
